# Integrating Indigenous Maya practices and digital health tools to improve outcomes for Indigenous newborns in Guatemala: a community-based initiative

**DOI:** 10.1080/16549716.2025.2565870

**Published:** 2025-10-06

**Authors:** Anahí Venzor Strader, Esteban Castro Aragón, Enma Coyote, Andrea I. Aguilar Ferro, Peter Rohloff

**Affiliations:** a Centro de Investigación en la Salud Indígena, Wuqu’ Kawoq, Tecpán, Guatemala; b Boston Children’s Hospital, Division of Emergency Medicine, Boston, MA, USA; c Division of Global Health Equity, Brigham and Women’s Hospital, Boston, MA, USA

**Keywords:** Guatemala, Indigenous, newborn health, home-based, digital health

## Abstract

Neonatal mortality remains a significant equity issue in rural Indigenous communities of Guatemala, where structural barriers and systemic discrimination impede access to quality newborn care. This field report describes a novel community-based initiative implemented by Maya Health Alliance, an Indigenous-lead NGO, to address high neonatal mortality in Maya Kaqchikel communities through a quality improvement (QI) framework. The intervention centers on home-based neonatal care delivered by trained Neonatal Technicians (NTs), supported by a co-designed smartphone application enabling early identification of neonatal danger signs, clinical decision-making, and data collection. The initiative also leverages a culturally responsive referral and patient navigation system to overcome humanistic barriers to care. Designed using QI methodology, the project applies iterative cycles to track key performance indicators such as perinatal and neonatal mortality rates, referral success rates, and the proportion of newborns receiving timely home evaluations. Since launching in 2024, the program has reached 85% of reported newborns, increased referral rates, and engaged local midwives and health staff through ongoing training and co-design efforts. However, challenges have emerged, including high prevalence of low birth weight, limitations in local hospital capacity, and discriminatory care at facilities that discourage families from accepting referrals. The intervention centers Indigenous practices by positioning TMMs at the frontline and adapting protocols to the communities’ lived realities. This initiative demonstrates the potential for culturally embedded, digitally supported, and equity-focused QI interventions to improve neonatal outcomes in resource-limited Indigenous settings. Future efforts will focus on expanding staff capacity, deepening community trust, and strengthening health system partnerships.

## Paper context

- **Main findings**: A community-based neonatal health program that integrates traditional Maya practices, home visits, and digital decision support in rural Guatemala has improved detection of at-risk newborns and referral rates.
- **Added knowledge**: This study highlights the feasibility of combining culturally tailored care, mHealth tools, and accompaniment in Indigenous settings, while documenting significant barriers rooted in structural inequities and cultural distrust.
- **Global health impact for policy and action**: Scaling such integrated models may strengthen neonatal care in marginalized communities, but it requires systemic reforms, investment in local health systems, and respect for cultural autonomy.

## Background

Neonatal mortality remains a pressing challenge worldwide, with 2.5 million newborn fatalities occurring annually [1]. Prematurity and low birth weight (LBW) are important drivers of excess neonatal deaths [2] and are more pervasive in resource-limited settings, such as the rural highlands of Guatemala. Although Guatemala’s reported neonatal mortality rate (10 per 1,000 live births) aligns with the Sustainable Development Goal (SDG) target [3], this statistic does not include stillbirths, which contribute greatly to perinatal mortality. Also, it does not fully capture the realities of rural communities, where many neonatal deaths go unreported [4,5].

Improving neonatal care in rural Indigenous Guatemala is a critical equity issue, as access to timely medical interventions in the perinatal period is significantly constrained [6,7]. Previous mixed-methods research in the region highlighted the compounding impact of structural forces such as poverty, geographic isolation, and systemic inequities on neonatal vulnerability and contributing healthcare system shortcomings [8]. Deep-seated mistrust of institutional healthcare, rooted in historical trauma and systemic neglect, strongly discourages families from seeking care, feeding a cycle of negative outcomes and worsening trust. However, collaborative relationships among public health facilities, traditional Maya midwives (TMMs) (We use this term throughout the article, as ‘Traditional Birth Assistant’, which does not accurately represent the societal role, cultural practices, and cosmovision of Maya midwives in Guatemala), and families have the potential to disrupt this cycle, foster trust, and improve outcomes [8].

For several decades, home-based neonatal care initiatives have gained recognition and demonstrated effectiveness in low-resource and rural settings. India has spearheaded the implementation of home-based neonatal care [9]. The cost-effectiveness of preventative and/or curative neonatal home visits and their efficacy in improving neonatal outcomes, rates of exclusive breastfeeding, and decreasing mortality are well documented [10–12]. These interventions are particularly helpful in rural settings with limited access to healthcare facilities. Interventions vary by setting, but commonly include home-based evaluations, detection of danger signs, and caregiver counseling by community health workers (CHWs). Some include at-home therapies for conditions typically requiring in-hospital care, such as neonatal sepsis, with promising outcomes [13,14].

The use of mobile health (mHealth) technology in resource-constrained settings to improve perinatal health outcomes has also gained popularity. Interventions include unilateral SMS-based communication with end-users sharing health information and advice, patient record-keeping, visit scheduling, patient or caregiver education, and decision-making support for health workers. Previous evidence regarding mHealth tools shows that they improve home visitation rates, early initiation of breastfeeding, healthcare coordination and quality, and data input. However, their impact on neonatal morbidity and mortality has not been thoroughly documented [15–19]. One systematic review noted that most studies evaluating mHealth tool efficacy on maternal, newborn, and child health (MNCH) outcomes are methodologically poor, and only a few evaluate direct patient outcomes [16]. Nonetheless, the potential of digital tools to improve access to maternal and newborn healthcare in remote and resource-constrained settings is significant.

Maya Health Alliance (MHA) is an Indigenous-led non-governmental organization that has implemented evidence-based, community-centered, and culturally appropriate initiatives for 20 years to improve health outcomes among Maya populations in Guatemala. Over 90% of MHA’s staff is Indigenous. Since 2015, co-developed an initiative with local TMMs to promptly identify high-risk obstetrical cases and refer them to advanced care using smartphone technology and low-cost monitoring devices [20]. Recognizing that technological advances are not inherently progressive and can perpetuate or exacerbate social inequities, we rely on co-design, close dialogue with communities, and cycles of praxis to ensure these tools advance health equity [21–23]. In 2024, MHA expanded this project to target newborns through home-based and mHealth-powered healthcare. To the best of our knowledge, home-based neonatal programs that incorporate mHealth initiatives have not been implemented with Indigenous populations in Latin America. This report describes a quality improvement initiative to enhance neonatal survival in Maya Kaqchikel communities in Guatemala through home-based interventions and mHealth technology.

## Brief description of the intervention

This intervention introduces home-based neonatal supervision by trained Neonatal Technicians (NTs), a specialized form of CHWs, who provide at-home comprehensive evaluations and support to mothers and caregivers. NTs use a co-designed smartphone application that facilitates early detection of neonatal danger signs and clinical decision-making. The intervention also incorporates clinical algorithms based on biomedical evidence and Indigenous practices. Our referral pathways are supported by health system navigators who address logistical and linguistic barriers, ensuring families receive timely, respectful, and compassionate care. The intervention, including the digital health component, has been shaped by an iterative co-design process with TMMs, NTs, and families’ feedback, positioning Indigenous knowledge as central rather than supplemental to biomedical practice.

## Context

This project is implemented in Tecpán, Guatemala by MHA, which has been transforming maternal healthcare since 2015 through a partnership with 40 local TMMs. These midwives play a pivotal role, attending around 700 births annually, with roughly half occurring at home. In collaboration with Emory University, Safe+Natal, and through a co-design process with TMMs, MHA developed a smartphone-based mHealth app to assist timely identification of obstetric complications [20,23]. MHA also established obstetric care navigators who accompany women to facilities, provide logistical support, interpretation, emotional assistance, and advocacy, helping overcome abusive or discourteous interactions and ‘humanistic barrier[s]’ to hospital care [24,25]. These initiatives have significantly improved maternal outcomes.

However, neonatal outcomes have not shown similar improvement. Previous efforts included incentivizing midwives to evaluate newborns and extending the Safe+Natal app for neonatal assessments and danger sign identification, but these were unsustainable and did not significantly reduce neonatal mortality [26]. MHA’s focus on integrating healthcare, research, and Indigenous language and culture is central to all its initiatives. By prioritizing community-based approaches and co-design, they ensure interventions are culturally appropriate, linguistically congruent, and responsive to Maya populations’ lived realities. This project builds on that foundation to address persistent gaps in neonatal survival through innovative, sustainable solutions.

## Study area and population

Tecpán, a municipality in Chimaltenango, has nearly 97,000 inhabitants, over 90% of whom identify as Maya Kaqchikel [27,28]. It spans 100 square miles of rugged terrain with over 40 rural villages, some requiring a 2.5-hour drive or accessible only on foot or by 4x4. As of 2023, 47.8% of Chimaltenango’s population lived in poverty, and 7.8% in extreme poverty [29]. According to the 2018 census, 54% of Tecpán’s population completed basic education, while only 2% attained higher education. Nearly 30% of women of reproductive age reported having their first child between ages 15 and 19 [28].

Tecpán’s local ‘Center for Medical Emergencies’ was upgraded to a level 1 hospital in 2018, expanding the services available for obstetrical and neonatal emergencies, such as the capacity to perform cesarean sections and mechanically ventilate neonates [30]. However, it lacks neonatal intensive care or subspecialty services and relies on referrals to hospitals in Chimaltenango (~40 min drive), Guatemala City (~2 hr drive), or beyond. A few private hospitals and birth centers offer maternity care, but serve only a small portion of the population due to cost. Other health services include rural posts, usually staffed by nurses or assistants, and health centers staffed by general practitioners and nurses.

This context partly explains the community’s reliance on TMMs for obstetric care and why nearly half of all deliveries occur at home. However, data from the Global Maternal and Newborn Health Registry [31] show institutional deliveries in Chimaltenango rose from 28.2% in 2010 to 57.7% in 2018 [32], partly due to improved roads, ambulance networks, and danger sign recognition [6,25].

## Quality improvement methodology and theory of change

The primary objectives of the present work are:

1. To reach 90% of newborns within our coverage area with an at-home comprehensive evaluation within the first 3 days of life by the first year.
2. To complete 90% of neonatal referrals successfully, including emergency and routine, within the first year of our program.
3. To decrease neonatal mortality rates by 50% over three years.

**Figure 1.**
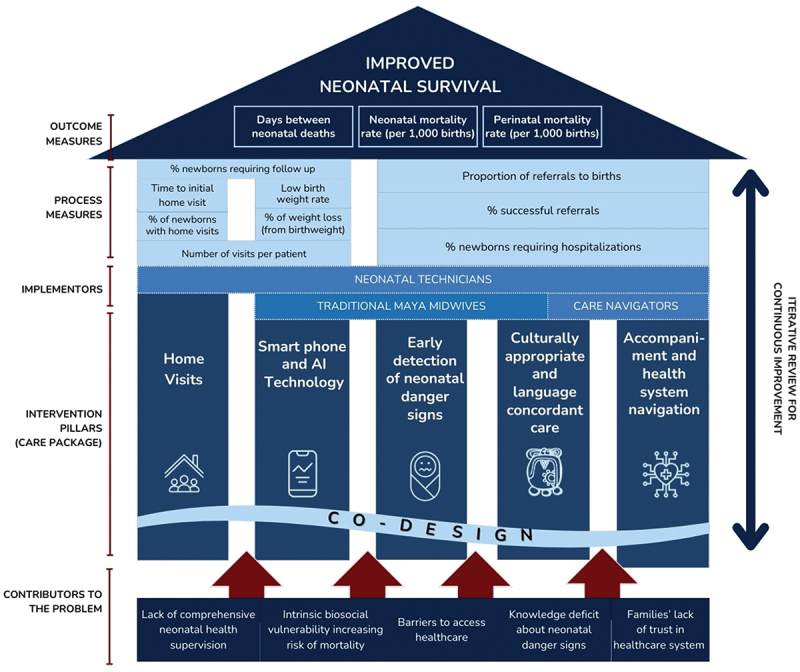
Neonatal health program’s theory of change.

This project’s theory of change focuses on mitigating the main drivers of excess neonatal mortality, which include inadequate supervision of newborns, biosocial vulnerabilities, and barriers to accessing healthcare through a multi-faceted intervention model (see Figure 1) centered on five pillars: home visits, early detection of neonatal danger signs, accompaniment, culturally appropriate care, and digital technology. Additionally, there is a strong emphasis on advocacy and co-designing interventions with multidisciplinary and multicultural teams. We employed a quality improvement (QI) methodology because it allows for rapid adaptations in response to dialogue with families and midwives, ensuring that the intervention remains responsive to community realities rather than bound by rigid research protocols.

Key performance indicators (KPIs) include perinatal and neonatal mortality rates, the proportion of newborns receiving timely home evaluations, neonatal referral rates, and referral success rates. The perinatal mortality rate is defined as the number of deaths occurring from 22 weeks of gestation, with a fetal weight 500 g or more, and up to 6 days of life (including intra and extrauterine deaths), per 1,000 births. The late neonatal mortality rate is defined as the number of deaths within days 7–28 of life per 1,000 live births. Additionally, a rare-events T-chart will track the interval between perinatal and neonatal deaths to identify improvement. We calculated our baseline mortality rates from the MHA internal birth registry; perinatal mortality is estimated as 24.4/1,000 births, and baseline late neonatal mortality as 3.8/1,000 live births from 2021 to 2023.

Data are extracted monthly. We analyze run charts to track KPIs over time, using established rules to detect non-random variation. Specifically, we consider a ‘shift’ as six or more consecutive points above or below the median and a ‘trend’ as five or more successive points increasing or decreasing, in line with standard quality improvement methodology. We align shifts and trends with intervention start points to ensure that KPI changes are attributable to the intervention. As balance measures, we track the number of home visits done monthly by NTs, the percentage of newborns visited in the first 3 days of life or post-hospital discharge, and the costs of our interventions. Routine checks, source verification, and periodic audits help ensure data completeness and accuracy. We also compare birth, stillbirths, and neonatal mortality rates with the municipality-level data as they make it available, particularly when sudden shifts appear.

## Detailed description of interventions

### Home newborn visits by neonatal technicians

We train and deploy NTs to perform at-home evaluations, identify neonatal danger signs, refer them to higher levels of care, and support caregivers with neonatal care. Their qualifications include a high-school diploma as a minimum, speaking Spanish and Maya Kaqchikel fluently, and living close to Tecpán. The community does not vote them in, as they cover a multitude of villages. However, they undergo a thorough selection process by the MHA human resources. Their responsibilities include home visitations, data recording in electronic medical records (EMR), coordinating hospital referrals, and communicating with families regarding care plans.

Every newborn has at least two home visits through the first month, starting within 3 days of birth if born at home or within the first 48 h of hospital discharge. The TMM who attended the birth or cared for the pregnant mother notifies the NT of the birth. Initial visits involve reviewing prenatal and antenatal data, newborn feeding and elimination patterns, presence of danger signs, and a physical examination that includes measuring rectal temperature, breathing rate, and weight. Newborns with LBW, difficulty feeding, or other conditions require more visits. The NTs also provide lactation support regarding latching, technique, and maternal/neonatal nutrition.

The training of NTs takes between 3 and 4 months. It involves:

1. 12–16 h of synchronous virtual teaching with lead pediatrician regarding the project’s objectives, communication skills, confidentiality, breastfeeding, neonatal physical exam, neonatal danger signs, and other relevant topics.
2. One week of in-person sessions with project administrators to teach data input, logistical task organization, internal and external referral processes, and other administrative tasks.
3. One to 2 weeks of in-person sessions to learn the use of the app and reinforce management protocols.
4. Observing multiple home visits accompanying TMMs in their prenatal visits and other neonatal technicians in postnatal visits.

Teaching and practice time is adapted based on individual learning needs and styles. Once training is completed, NTs can conduct supervised home visits before transitioning to independent visits. NTs are MHA’s employees and not part of the official health workforce. However, they collaborate closely with rural health post staff, coordinating follow-ups and referral logistics.

The smartphone app streamlines neonatal care by facilitating communication and data collection. It offers clinical guidance, medical history tracking, and communication tools. Throughout their participation in the program, NTs have contributed to the design and improvement of the app and EMR formularies. They have adapted home visit procedures to cultural expectations while preserving the quality of care. Clinical algorithms responding to danger signs were established by physicians and nurses based on global clinical evidence and adapted to local cultural practices and resources available based on NT’s, TMM’s, and families’ feedback.

### Smartphone mHealth technology

We co-designed a smartphone app that gathers clinical data, provides real-time feedback on metrics, and guides clinical decision-making without requiring an active internet connection. The app interprets inputted physiologic data and, for example, instructs the technician to place the baby skin-to-skin with the mother if the temperature is low and to recheck in 20–30 min. It also indicates whether the birth weight is adequate, low, or very low, calculates the weight change since birth, and determines whether percentage of weight loss or daily weight gain is acceptable. The NTs’ input has been invaluable during the app co-design process.

The app also has a checklist of danger signs with images and brief descriptions. When a danger sign is present, the app guides management based on previously established clinical algorithms and offers the option to call the medical team with questions or the on-call navigator to coordinate referral. When concluding the visit, the app sends a text message to the NT with deidentified collected information to facilitate inputting this data into the EMR.

### Continuous education and support to midwives

Enhancing the TMMs’ skillset to provide newborn care is imperative to strengthen the maternal-child healthcare infrastructure in the region. Therefore, we regularly conduct hands-on training sessions for the collaborating midwives. We conduct Essentials of Newborn Care courses [33] and equip them with neonatal resuscitation equipment, such as upright ventilators and airway suction devices. Additionally, we have provided all TMMs with digital hanging scales and baby carriers to ensure uniformity in birth weight data and co-developed an easy way of inputting this data into the Safe+Natal app.

Other educational sessions focused on the detailed neonatal physical exam to identify congenital abnormalities and the care of the small and vulnerable baby (LBW and/or premature), including hands-on practice on passive feeding and Kangaroo-Mother-Care (KMC) techniques. All trainings were held in person with live translation to Maya Kaqchikel. Additionally, program coordinators visit the TMMs bimonthly to reinforce the use of the app and help troubleshoot any technology issues.

### Integration with the patient navigation system

MHA’s obstetrical care navigation program has been described elsewhere [25]. We leverage their services to support our families through neonatal referrals. Navigators receive notification of routine or emergency referrals from TMMs and NTs. They coordinate transportation by ambulances or private vehicles, accompany families to the hospital, and stay with them until the baby is either discharged or admitted. Additionally, they provide interpretation for Maya Kaqchikel speakers and assist caregivers with purchasing medications or paying for diagnostic studies as necessary. They also communicate regularly with families via phone to ensure they can attend follow-up appointments and follow discharge instructions.

### Culturally relevant care

Following MHA’s commitment to elevate the value of Indigenous Maya languages and culture in healthcare, we consider culturally relevant care as one of our intervention’s pillars. Maya midwives are frontline health providers, advocates, and community leaders, and their centuries-old practices shape the foundation of this program. From the outset of the MHA maternal health program, we collaborated with TMMs to co-design tools and care. This approach ensures that biomedical tools complement and do not displace existing knowledge. The present neonatal intervention’s design stems from mixed-methods research conducted in collaboration with TMMs and patients, which clearly demonstrated the need for a home-based intervention. All prenatal and postnatal visits are conducted in our patients’ preferred language, which in the great majority of cases is Maya Kaqchikel. We respect families’ preference for home births and their apprehensions about hospital care, which arise from past experiences of discrimination and adverse encounters. This reflects our commitment to positioning families as decision-makers rather than passive recipients of external directives. To center Indigenous expertise, we incorporate ancestral knowledge shared by TMMs, such as plants used to boost breastmilk production, home-based methods to care for small and vulnerable newborns, and others. For example, families prefer the *temazcal* (Maya sauna) for bathing and to prevent hypothermia. Therefore, we are co-developing protocols with TMMs for their safe use to prevent overheating and other complications. By doing so, we position Indigenous realities and agency at the forefront, moving beyond a biomedical framework to a model of shared practice and mutual respect.

## Early outcomes and impact

**Figure 2.**
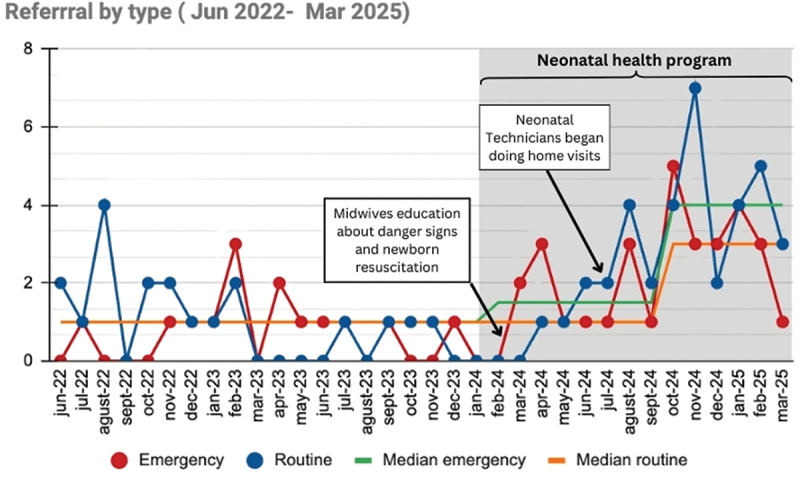
Runchart of emergency and routine neonatal referrals.

Since our project began in January 2024, to March 2025, 839 births have been reported. From July 2024, when NTs were deployed, 270 neonates have been visited. We have reached 85% of the newborns reported monthly, although only 30% have been visited during the first 3 days of life (see the characteristics of the patients visited in Table 1). The referral rate has increased by up to 200% (see Figure 2). The current routine referral rate is 10%, while the emergency referral rate is 4%, with a successful completion rate of 63% and 77%, respectively. The most common referral indications are LBW and feeding difficulties. The proportion of babies with LBW (under 2,500 g) is 24.8%. There have been 17 stillbirths and 15 neonatal deaths since January 2024, constituting a perinatal mortality rate of 26.2 per 1,000 births and a neonatal mortality rate of 8.5 per 1,000 live births.

**Table 1.** Characteristics of newborns visited by neonatal technicians from July 2024 to March 2025.

Variable		n	%
Age at first visit, days			
0–3		72	26.7
4–7		67	24.8
8–14		68	25.2
15–30		56	20.7
>30		7	2.6
Birth method			
Vaginal delivery		177	66.0
C-section		91	34.0
Birth attendant			
Traditional maya midwife		137	51.1
Physician		128	47.8
Nurse or other		3	1.1
Birth location			
Home		136	50.6
Hospital		130	48.3
Other		17	6.0
Reported gestational age			
>37 weeks		227	88.7
<37 weeks		29	11.3
Birth weight classification			
Normal		206	76.3
Low birth weight <2,500 g		61	22.6
Very low birth weight, <1,500 g		3	1.1
Feeding method			
Exclusive breastfeeding		234	86.7
Mixed feeding		34	12.6
Formula		1	0.4
Other		1	0.4
Time to first breastfeeding			
Less than one hour		157	59.5
1–4 hours		67	25.6
More than 4 hours		40	15.2
Neonatal Danger signs identified			
None		155	59.2
Excessive crying		2	0.8
Neonatal asphyxia		1	0.4
Respiratory distress		1	0.6
Fever		4	1.5
Excessive or inadequate weight loss		18	6.9
Jaundice		2	0.8
Prematurity		12	4.6
Umbilical stump infection		1	0.4
Low birth weight		65	24.8
Prenatal complication			
None		183	67.8
Postpartum hemorrhage		2	0.7
Headache		5	1.9
Fever		1	0.4
History of elevated BP		4	1.5
Abnormal fetal presentation		1	0.4
Elevated blood pressure		18	6.7
Premature labor		4	1.5
Other		41	15.2

## Challenges and barriers

### Technical challenges

The digital hanging scales’ accuracy is substandard, requiring a shift to alternative devices for more precise measurements. Connectivity and phone signal in the villages are unreliable and limit effective communication. Reaching patients’ homes via public transportation can take several hours, significantly reducing the number of home visits that NTs can perform daily. The institution lacks dedicated vehicles and drivers for our program.

### Human resources

Workforce constraints pose significant challenges for timely and comprehensive newborn care. Recruitment of new NTs has been challenging, given that structural factors such as gender inequity and chronic disinvestment in Indigenous and rural communities limit educational opportunities for women in the region. With a limited number of NTs, our ability to provide as close follow-up as intended is limited. In response, we have increasingly relied on the navigators for accompaniment to routine referrals, phone follow-ups, and documentation efforts.

### Community-level and cultural challenges

Cultural perceptions and customs have influenced newborn care practices. Many families are hesitant to have their newborns undressed for weighing and physical examination. The acceptance and implementation of KMC has also been challenging due to modesty norms and because traditional attire is not suitable for it. Hesitation to both routine and emergency referrals is significant, and stems mostly from fear of separation from their newborn and previous adverse experiences at hospitals. Common accounts of discrimination and mistreatment toward Indigenous people at local hospitals contribute to this hesitation. When the indication for referral is serious or urgent, our approach focuses on providing clear, evidence-based guidance so caregivers can make an informed decision. Additionally, we follow up with patients by phone and communicate with local health post staff to ensure their awareness of these cases.

Rather than viewing resistance to referrals as a barrier, we recognize it as families exercising agency and preserving their cultural tradition of keeping mom and baby at home during the newborn period. Therefore, we adapted our protocols to expand the in-home care for small babies by teaching passive feeding techniques, supporting lactation through breast pumps and midwife-recommended herbs, educating caregivers about danger signs, and increasing home visits when feasible. Additionally, we developed algorithms and trained NTs to detect and treat minor conditions such as oral or diaper candidiasis, diaper dermatitis, dacryostenosis, and umbilical granuloma. Through these adaptations, we aim to integrate local practices into our care.

### Institutional barriers – health system deficiencies

The local hospital has limited capacity to manage complex neonatal and, at times, numerous referrals. As a result, transfers to larger regional hospitals have been necessary, though these facilities also face resource limitations, leading to care delays. To address this, we work closely with in-hospital providers and health authorities to adapt our procedures, communicate about high-risk cases, and increasingly leverage the services of rural health posts and community health centers. However, rural facilities are inconsistently staffed, often by personnel with limited training, resulting in variable care quality. In response, we optimize the care of the small baby at home and offer training for rural health post staff in coordination with local authorities regarding the management and monitoring of small and vulnerable babies in the community. Other significant barriers are the lack of Maya Kaqchikel-speaking providers in the hospital and the overall devaluation of Indigenous practices. Patients are commonly scorned for seeking care from TMMs and using *temazcales* (Maya sauna baths) and other ancestral practices that are central to community health. These factors strongly impact the families’ denial of referrals.

## Discussion

In this paper, we describe a novel community-based intervention aimed at improving neonatal outcomes in Indigenous communities in rural Guatemala through home visits, integration of Indigenous practices, and digital technology. Preliminary results demonstrate improvements in referral rates and success within the first year, with observed changes appearing non-random and temporally aligned with our interventions. Perinatal and late neonatal mortality rates measured in 2024 are higher than our 2021–2023 baseline. We believe this increase largely reflects improved monitoring of neonatal outcomes, leading to more accurate detection of fatalities that may have previously gone uncounted. While some rise was anticipated, the magnitude of the increase in stillbirths, more common among first-time mothers, is particularly concerning. We have initiated further investigations to explore additional possible explanations for this phenomenon. In collaboration with our colleagues at local hospitals, we analyzed their data and discovered a comparable rise in the incidence of stillbirths in 2024 compared to the preceding 3 years (unpublished data). We are also following primiparous pregnancies more closely and encouraging hospital births in these cases. More research is needed to understand the factors driving this increase in stillbirths.

Notably, the prevalence of LBW exceeds previously reported rates in the region [34] and accounts for the majority of our referrals, leading to an overload of local healthcare resources. Consequently, we are prioritizing improving the care of the small baby at home. Previous studies have evaluated the implementation of thermal care and KMC at home with varied results, but overall, promising efficacy of KMC to improve outcomes [35]. However, qualitative reviews of home-based neonatal hypothermia prevention reveal that the uptake of these practices can be challenged by strong cultural norms and traditions, postpartum pain, fear of harm, time constraints, lack of back support, or breastfeeding issues [35,36].

Other notable dynamics we have encountered include caregivers’ hesitations to some aspects of care. A qualitative study on a home-based neonatal care intervention by Community Health Workers (CHWs) in India revealed similar challenges. Their strategies included adapting their counseling techniques to the cultural context, balancing compliance with traditional practices while promoting evidence-based techniques, and occasionally deviating from traditional practices, particularly during emergencies [37]. Furthermore, evidence from more experienced home-based neonatal care programs suggests that regular assessments of CHWs to identify needs and challenges, and continuous education to fill knowledge gaps, are imperative to maintain positive outcomes [38].

Unlike many mHealth initiatives that primarily digitize biomedical care, this project is distinct in that community practices directly inform our protocols. For example, in dialogue with TMMs, we lowered the threshold for referral of low birth weight newborns to reduce the risk of mother – baby separation and adhere to quarantine practices that emphasize keeping mothers and infants together at home. Additionally, we have started treating minor conditions such as neonatal candidiasis at home to prevent families from leaving the household during this sensitive period. These adaptations illustrate how QI cycles enable the integration of Indigenous knowledge and practices into service delivery, positioning community realities not as obstacles to overcome but as guiding principles of care. We recognize that when families choose to decline referrals despite the presence of danger signs, neonates may face increased risk for adverse outcomes. This tension is concerning and requires careful navigation. However, we consider respecting families’ agency to be essential for building value-based care models grounded in decolonizing practices. Our experience suggests that healthcare models relying on coercion erode trust and ultimately perpetuate inequities [8].

Some of the barriers we encountered reflect deeper structural inequities, including long-standing disinvestment in Indigenous and rural communities, and pervasive racism and ethnic discrimination. Healthcare facilities in Tecpán are unable to meet the high demand or provide consistent, quality care due to the centralization of Guatemala’s public health system, which prioritizes Guatemala City and leaves rural centers severely under-resourced. These disparities are rooted in colonial legacies and reinforced by Western-centric ideologies that marginalize Indigenous languages and ways of life. These legacies were made starkly visible during the civil war and the genocide of Maya people [39]. As a result, families’ reluctance to seek in-hospital care represents a complex, historically grounded distrust of state institutions, an effort to exert agency, and a resistance to cultural erasure [40].

Limitations of this work include its preliminary status. Consequently, no final conclusions can be drawn at this stage, as we continue to learn from the intervention. Additionally, the design and initial results are highly influenced by the local context, which limits the generalizability of our findings. Nonetheless, we present a feasible, community-based, and innovative approach to improving neonatal outcomes in a rural, low-resource setting. Ongoing data analysis and iterative adjustments are underway to assess the intervention’s impact on mortality.

## Future directions

In 2025, we are expanding our team of NTs, patient navigators, and TMMs to increase program reach. Continuous training is also foreseen to empower TMMs and NTs with increased capacity to respond to the challenges faced. In a parallel project, MHA is also integrating artificial intelligence (AI) into the Safe+Natal app to predict fetal growth restriction and preeclampsia from Doppler fetal heart recordings, enhancing early detection and timely intervention [41]. Given the high prevalence of LBW, we plan to include other home-based care strategies such as novel low-tech baby warmers [42], KMC carriers, and passive feeding supplies (manual breast pumps and feeding cups). Finally, strengthening our partnerships within the health system remains a key priority. We continue to offer training on essential neonatal health topics for local health posts staff and are working towards co-developing evidence-based guidelines for common neonatal conditions.

## Conclusion

We present a community-based intervention for neonates in Maya communities of rural Guatemala, integrating Indigenous care practices through collaboration with Maya midwives, mHealth technology via a smartphone-based clinical decision support app, and accompaniment principles through home visits and support navigating the healthcare system. The intervention centers Indigenous knowledge and practices by positioning TMMs at the frontline and adapting protocols to reflect the communities’ lived realities. This quality improvement initiative shows early, promising results, with increased rates of both emergency and routine referrals. These preliminary results indicate the potential of this intervention to positively impact neonatal outcomes. However, several challenges have emerged, including an unexpectedly high prevalence of LBW, staffing shortages, limited capacity at referral centers, and caregivers’ reluctance to hospital referrals due to fear of negative experiences. These barriers are shaped by structural violence, including the long-standing disinvestment in rural health infrastructure and persistent ethnic discrimination.

## Supplementary Material

SQUIRE_checklist.docx

## Data Availability

De-identified data described in the manuscript, code book, and analytic code will be made publicly and freely available upon publication without restriction at: Strader, Anahi, 2025, ‘Replication Data for: Integrating Traditional and Digital Health to Improve Outcomes for Indigenous Newborns in Guatemala: A Community-Based Initiative’, https://doi.org/10.7910/DVN/VF8YAW, Harvard Dataverse.
